# Interfering With DNA Decondensation as a Strategy Against Mycobacteria

**DOI:** 10.3389/fmicb.2018.02034

**Published:** 2018-09-05

**Authors:** Enzo M. Scutigliani, Edwin R. Scholl, Anita E. Grootemaat, Sadhana Khanal, Jakub A. Kochan, Przemek M. Krawczyk, Eric A. Reits, Atefeh Garzan, Huy X. Ngo, Keith D. Green, Sylvie Garneau-Tsodikova, Jan M. Ruijter, Henk A. van Veen, Nicole N. van der Wel

**Affiliations:** ^1^Electron Microscopy Center Amsterdam, Academic Medical Center, Amsterdam UMC, University of Amsterdam, Amsterdam, Netherlands; ^2^Medical Biology, Academic Medical Center, Amsterdam, Netherlands; ^3^Department of Pharmaceutical Sciences, University of Kentucky, Lexington, KY, United States

**Keywords:** *Mycobacterium tuberculosis*, antibiotic, DNA condensation, high resolution analysis, Eis inhibitor

## Abstract

Tuberculosis is once again a major global threat, leading to more than 1 million deaths each year. Treatment options for tuberculosis patients are limited, expensive and characterized by severe side effects, especially in the case of multidrug-resistant forms. Uncovering novel vulnerabilities of the pathogen is crucial to generate new therapeutic strategies. Using high resolution microscopy techniques, we discovered one such vulnerability of *Mycobacterium tuberculosis*. We demonstrate that the DNA of *M. tuberculosis* can condense under stressful conditions such as starvation and antibiotic treatment. The DNA condensation is reversible and specific for viable bacteria. Based on these observations, we hypothesized that blocking the recovery from the condensed state could weaken the bacteria. We showed that after inducing DNA condensation, and subsequent blocking of acetylation of DNA binding proteins, the DNA localization in the bacteria is altered. Importantly under these conditions, *Mycobacterium smegmatis* did not replicate and its survival was significantly reduced. Our work demonstrates that agents that block recovery from the condensed state of the nucleoid can be exploited as antibiotic. The combination of fusidic acid and inhibition of acetylation of DNA binding proteins, via the Eis enzyme, potentiate the efficacy of fusidic acid by 10 and the Eis inhibitor to 1,000-fold. Hence, we propose that successive treatment with antibiotics and drugs interfering with recovery from DNA condensation constitutes a novel approach for treatment of tuberculosis and related bacterial infections.

## Introduction

Tuberculosis (TB), caused by *Mycobacterium tuberculosis* infection, is the leading cause of death from an infectious disease, resulting in 10.4 million new cases world-wide, including around 500,000 humans infected by the multi-resistant form, and an estimated 1.4 million deaths in 2016 alone (WHO, 2017). In recent years, multidrug-resistant, extensively drug-resistant and totally drug-resistant *M. tuberculosis* strains have emerged, and in some regions the percentage of patients infected by multidrug-resistant tuberculosis is well above 50% (Zignol et al., 2016). Thus, new therapeutic approaches are urgently needed. Since part of the tuberculosis casualties are caused by the reactivation of *M. tuberculosis* in granuloma of latently infected individuals (Dye and Williams, 2010), strategies for treating the latent form of this disease are essential.

This study focuses on the preservation of bacterial genome integrity by the temporary condensation of chromosomal DNA, a process demonstrated to occur during latency and other stressful conditions in *Escherichia coli* (Wolf et al., 1999), *Bacillus subtilis* (Smith et al., 2002), *Helicobacter pylori* (Ceci et al., 2007), cyanobacteria (Murata et al., 2016), and *Deinococcus radiodurans* (Eltsov and Dubochet, 2005). This phenomenon was observed for the first time during electron microscopic imaging of starved *E. coli*, and led to the hypothesis that a compact structure of DNA and DNA-binding proteins might act as a physical barrier (Wolf et al., 1999; Smith et al., 2002; Qu et al., 2013). Stress-induced DNA condensation was also shown to promote homology-driven repair of DNA double strand breaks in *E. coli*, further demonstrating a role for nucleoid-condensation in maintaining genome integrity during stress (Shechter et al., 2013). Moreover, DNA condensation is thought to be associated with quiescence (Rittershaus et al., 2013), and quiescent mycobacteria are able to condense their DNA (Wu et al., 2016a,b,c). Interestingly, quiescent *M. smegmatis* with condensed nucleoids display reduced metabolism and increased tolerance to stress and antibiotics (Wu et al., 2016c).

The mechanisms driving nucleoid-condensation remain to be identified. Computational simulations of idealized DNA structural monomers showed that attraction is sufficient to collapse a chain of large structural DNA monomers and that entropic forces exerted by molecular crowding can cause compaction of chromosomes (Pelletier et al., 2012; Shendruk et al., 2015). These computational modeling studies combined with physical manipulation demonstrated that *E. coli* chromosome behaves as a loaded entropic spring *in vivo*. Besides the entropic forces, this process also involves nucleoid-associated proteins. These nucleoid-associated proteins (NAPs) are homologous to histones and especially well studied in *E. coli*. Here, NAPs interact with DNA and influence replication, transcription and compaction (reviewed by Dillon and Dorman, 2010). As mycobacterial NAPs have limited homology to *E. coli* NAPs, a number of mycobacterial DNA binding proteins have been identified as NAPs only recently. HupB was described to be binding to the origin of replication in *M. smegmatis* (Hołówka et al., 2017). Deletion of Rel was shown to affect the lipid and DNA distribution in *M. smegmatis* (Wu et al., 2016a). Also in *M. smegmatis*, overexpression of histone like protein H-NS or Rv3852 from *M. tuberculosis*, results in a less compact nucleoid morphology (Ghosh et al., 2013). In addition, the change in the acetylation state of the nucleoid associated proteins can cause a shift in the DNA localization in this microorganism (Ghosh et al., 2016).

Here we report that DNA condensation is a physiological response to antibiotic stress or starvation conditions in the clinically relevant *M. tuberculosis*. We also demonstrate that, under normal conditions, this response is reversible, as DNA returns to the initial decondensed state after withdrawal of the stress-inducing agent. Importantly, however, blocking this decondensation step by inhibition of histone-like protein acetylation, sensitizes bacteria to the stress-inducing agent and dramatically reduces their survival. These results uncover a previously unknown vulnerability of *M. tuberculosis*, which can be exploited for the development of conceptually novel treatment strategies. Moreover, as stress-induced nucleoid condensation is being recognized as a common response in bacteria, we propose that rationally designed antibiotics regimens targeting changes in DNA condensation state can form the basis for broadly applicable antibacterial approaches.

## Materials and methods

### Bacterial strains and culturing conditions

*M. smegmatis* mc^2^155, *M. smegmatis* mc^2^6, *M. smegmatis* mc^2^3449 structural maintenance of chromosomes (SMC) triple deletion mutant (Gifts from Paras Jain and William R Jacobs, unpublished and Panas et al., 2014) and *M. tuberculosis* mc^2^6030 (originally described in Sambandamurthy et al., 2002) were grown in Middlebrook 7H9 medium supplemented with 0.05% Tween-80, 0.2% glycerol, and 10% Oleic Albumin Dextrose Catalase (OADC) at 37°C while shaking to an OD_600_ ranging between 0.1 and 0.6 at the start of the experiment. Pantothenate-auxotroph strain mc^2^6030 was supplemented with 24 μg/ml pantothenate. For starvation experiments, cultures were grown in PBS/Tween-80 at 37°C while shaking.

### Antibiotic solutions

Bacteria were subjected to 20 times the MIC_50_ of fusidic acid (FA) (250 μg/ml; Sigma-Aldrich), nalidixic acid (330 μg/ml; Sigma-Aldrich), isoniazid (2 μg/ml; Sigma-Aldrich), linezolid (20 μg/ml; Sigma-Aldrich), streptomycin (20 μg/ml; SERVA), rifampicin (20 μg/ml; Sigma-Aldrich), Eis inhibitor 1a^*^ (10 μM) (Chen et al., 2012; Green et al., 2018), or DMSO (solvent control; Merck Millipore) for 1 h.

### Transmission electron microscopy and tomography

*M. smegmatis* mc^2^155 or *M. tuberculosis* mc^2^6030 was subjected to FA for 1 h, fixed with McDowell fixative in 0.1 M sodium cacodylate buffer and postfixed with kaliumhexacyanoferrate (VWR) and 1% osmiumtetroxide (Electron Microscopy Sciences) in cacodylate buffer. Samples were embedded in gelatin and after ethanol dehydration, embedded in Epon (Ladd Research). Grids covered with formvar were used to collect 50-80 nm sections made using a Leica EM FC6 (Leica). Sections were stained using uranyl acetate and lead citrate. Electron microscopy images were collected using a FEI Tecnai™ transmission electron microscope with a LaB_6_ filament (Denka) at 120 kV. For tomography, 100/200 nm thick sections of epon embedded *M. smegmatis* with or without FA were imaged with ±60° tilt series, with 5° increments. Images were aligned using Fourier filtered cross correlation and reconstructed by SIRT (Simultaneous Iterative Reconstruction Technique) with 25 iterations using the Inspect3D Xpress software.

### Fluorescence microscopy and combined light and electron microscopy

For fluorescence microscopy on fixed samples, cultures were fixed by resuspension in fixative with paraformaldehyde and glutaraldehyde (Sigma-Aldrich) for 4 h. Next, fixed bacteria were transferred to storage buffer with paraformaldehyde. DNA was visualized by Hoechst 33342 (Thermo Fisher). Cell membranes and lipid inclusions were visualized by either BODIPY® 558/568 C_12_ (4,4-difluoro-5-(2-thienyl)-4-bora-3a,4a-diaza-*s*-indacene-3-dodecanoic acid; Molecular Probes) or Nile Red (9-diethylamino-5H-benzo[a]phenoxazine-5-one; Sigma-Aldrich) for 5 min in the dark at room temperature. After incubation, coverslips were mounted by using VectaShield Mounting Medium (Vector Laboratories). Wide-field fluorescence microscopy images were collected using a Leica DM-RA light microscope equipped with a 100x Plan Apo 1.4 Phaco3 oil-immersion objective lens. Confocal fluorescence microscopy images were collected using a Leica SP8-X SMD confocal fluorescence microscope fitted with a 63x Plan Apo NA 1.4 CS3 oil-immersion objective lens (Leica). Excitation of fluorophores was done using a 100 mW White Light Laser (Leica) and detected using a variable bandpass filter (Leica) with HyD or photomultiplier tube detectors (Leica). For CLEM, fixed *M. smegmatis* mc^2^155 cultures were stained for DNA and lipids and incubated on Carbon coated golden reference finder grids (Electron Microscopy Sciences). Grids were subsequently analyzed with confocal fluorescence microscopy and Transmission Electron Microscopy, and the images were matched using Photoshop.

### Monitoring mycobacterial survival

*M. smegmatis* mc^2^155 overnight cultures were treated with FA, Eis inhibitor 1a^*^(Chen et al., 2012; Green et al., 2018), or a combination hereof. Cultures were treated with antibiotic for 1 h before second antibiotic was administered. Before treatment, and after 2 h and at day 1, 2, 3, and 6, the OD_600_ was determined with a spectrophotometer, and a sample of the culture was fixed, or plated in a dilution series on 7H10 plates in triplicate. At day 2, the antibiotic treatment was repeated. At least 3 independent experiments were quantified per condition.

### Live cell imaging

For live cell imaging, bacteria were stained with the LIVE/DEAD® *Bac*Light® Bacterial Viability Kit. *M. smegmatis* mc^2^155 *or M. tuberculosis* mc^2^6030 suspension was incubated on poly-L-lysine-coated multiwell microscopy slides in the dark at room temperature. FA was added before coverslips were mounted, and bacteria were monitored using wide-field fluorescence microscopy. *M. smegmatis* cultures were treated with FA and time-lapse images (time interval of 2 or 5 min) were acquired using a fully motorized Leica DMi8 inverted widefield fluorescence microscope (Leica Microsystems, Wetzlar, Germany) equipped with culture incubator. Images were recorded with a high numerical aperture 63 × oil immersion objective (HC PL APO CS2 63.0 × 1.40 OIL UV; Leica Microsystems) [immersion oil, Leica Type N, n_D_(refractive index) = 1.518 (at 23°C); Leica Microsystems, Wetzlar, Germany] using a 16-bit Hamamatsu ORCA–Flash4.0 V2 sCMOS C11440-22CU camera (Hamamatsu Photonics, Hamamatsu, Japan) with Leica Application Suite X image acquisition software and a GFP filter (Ex: 450-490 nm, Dc: 495 nm, Em: 500-550 nm; Leica Microsystems, Wetzlar, Germany). After deconvolution from ~4 to 5 z-sections with 0.5 μm spacing, images were analyzed by local background subtraction and thresholding using Huygens Software (Scientific Volume Imaging, SVI, Hilversum, The Netherlands). Final image adjustments were done using ImageJ 1.49s (National Institutes of Health, Bethesda, MD).

### DNA volume quantification

Z-stack Wide-field FM images of fixed *M. smegmatis* mc^2^ 155 stained for DNA and lipids were collected at 200 nm increments. Deconvolution and DNA volume quantification was subsequently performed using standard deconvolution parameters of Huygens Professional software (Scientific Volume Imaging, SVI, Hilversum, The Netherlands). Average volume was determined for 60 bacteria per condition per experiment and standard deviation was calculated with 2-tailed *t*-test.

### Statistical analysis CFUs

In all experiments, factor correction (Ruijter et al., 2006) was applied to remove systematic differences between the different measuring sessions needed to obtain the results. In case of 2 experimental conditions, Student's *t*-test was applied. More than 2 conditions were compared with 1-way Analysis Of Variance (ANOVA). When more conditions were compared at different time points, a 2-way ANOVA always showed a significant interaction between culture conditions and time points, indicating that the effects of the culture conditions dependent on the time of analysis. To further dissect these effects, a 1-way ANOVA per time point was performed. After each 1-way ANOVA a *post-hoc* Student-Newman-Keuls was applied to determine subsets of conditions with similar effects; conditions in different subsets differ significantly from each other. *P*-values < 0.05 were considered significant.

## Results

### Stress-induced ultrastructural rearrangement of DNA in *M. smegmatis* and *M. tuberculosis*

**Figure 1:**
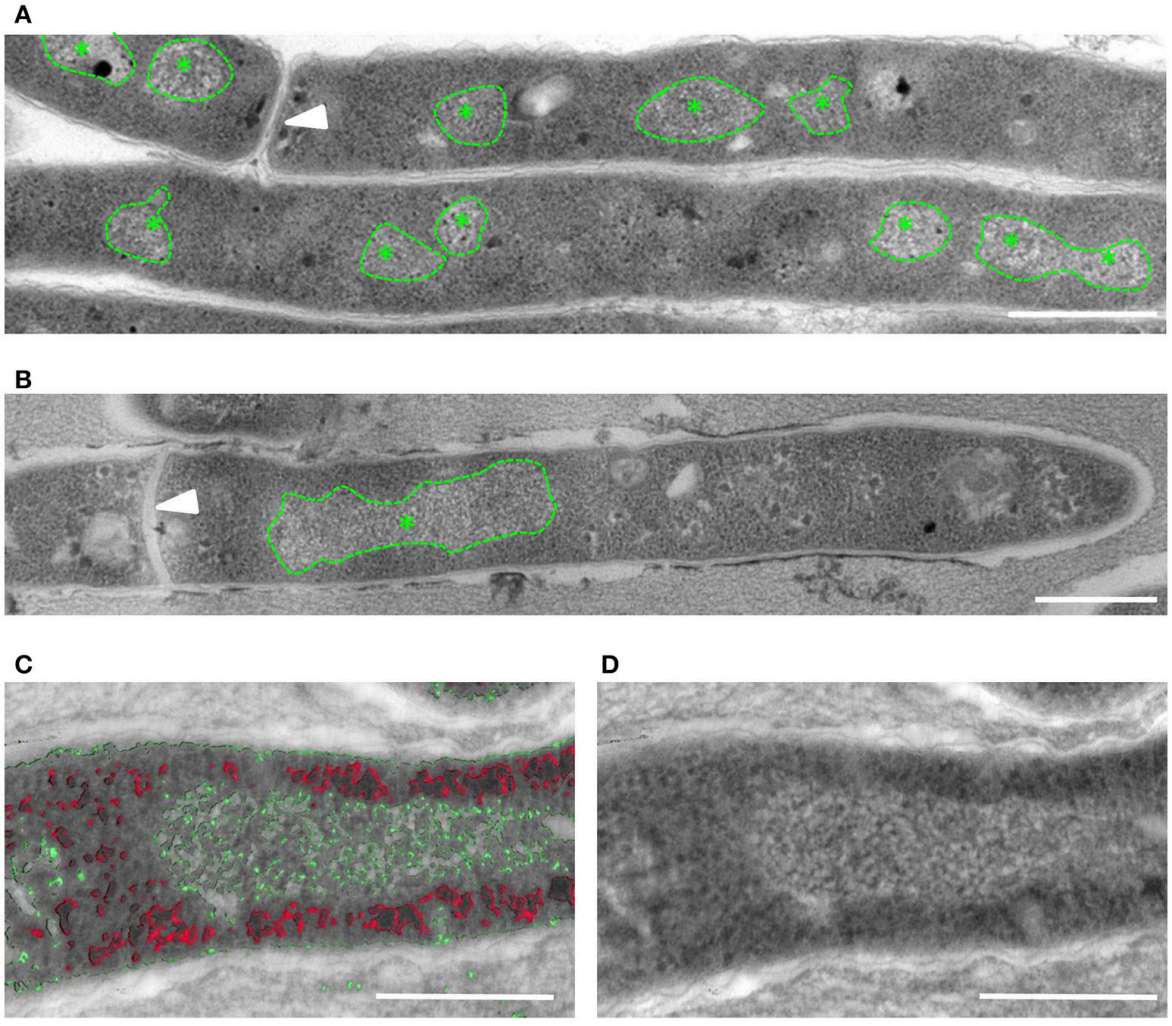
Altered localization of DNA visible in M. smegmatis after FA treatment. TEM-images of ultrathin (80 nm) sections of untreated **(A)** and FA-treated **(B)** *M. smegmatis* showing a single, enlarged area with typical DNA structure (*) in the FA treated bacteria. **(C,D)** Tomogram slices containing a semi-thick (300 nm) section of FA treated *M. smegmatis*. Stacks are artificial color coded based on electron-density, with in red e-dense ribosome-like structures, in green DNA clusters **(C)**, and the separate TEM image **(D)**. Arrowhead indicates septum, * indicates typical DNA structure and all scale bars represent 500 nm.

Previous studies using transmission electron microscopy (TEM) have identified striated bundles of crystalline DNA in different bacterial species (Levin-Zaidman et al., 2000; Eltsov and Dubochet, 2005). To determine if mycobacteria undergo similar ultrastructural changes, TEM was performed on ultrathin sections of early log phase *M. smegmatis* mc^2^155 control cultures (OD < 0,8) and cultures treated with the antibiotic fusidic acid (FA). FA inhibits protein synthesis by blocking GTPase activity of ribosomal elongation factor G. In FA treated *M. smegmatis*, bundles of DNA were localized in a single compact nucleoid, whereas DNA in untreated bacilli was more dispersed in multiple smaller nucleoids (Figures 1A,B). To enhance the resolution of smaller ultra-structures such as DNA and ribosomes, tomographic analysis was performed on semi-thick sections of Epon-embedded *M. smegmatis*. Using differences in (electron) density, we were able to identify enlarged, striated clusters of DNA (in green), which did not overlap with ribosomes (in red, Figures 1C,D and Supplemental Figures 1A,B).

To confirm that the induced rearrangements indeed involve DNA, we performed Correlative Light and Electron Microscopy (CLEM). To this end, intact *M. smegmatis* were fixed and applied to finder grids to enable tracing individual bacteria first using fluorescence microscopy (FM) and subsequently with EM. CLEM analysis demonstrated that the electron-lucent, ribosome-free clusters co-localized with the fluorescence signal of DNA in the bacteria (Supplemental Figures 1C,D). Taken together, these data indicate that nucleoid condensation in distinct areas takes place in *M. smegmatis* upon FA treatment.

### DNA condensation is a general stress response in mycobacteria

**Figure 2:**
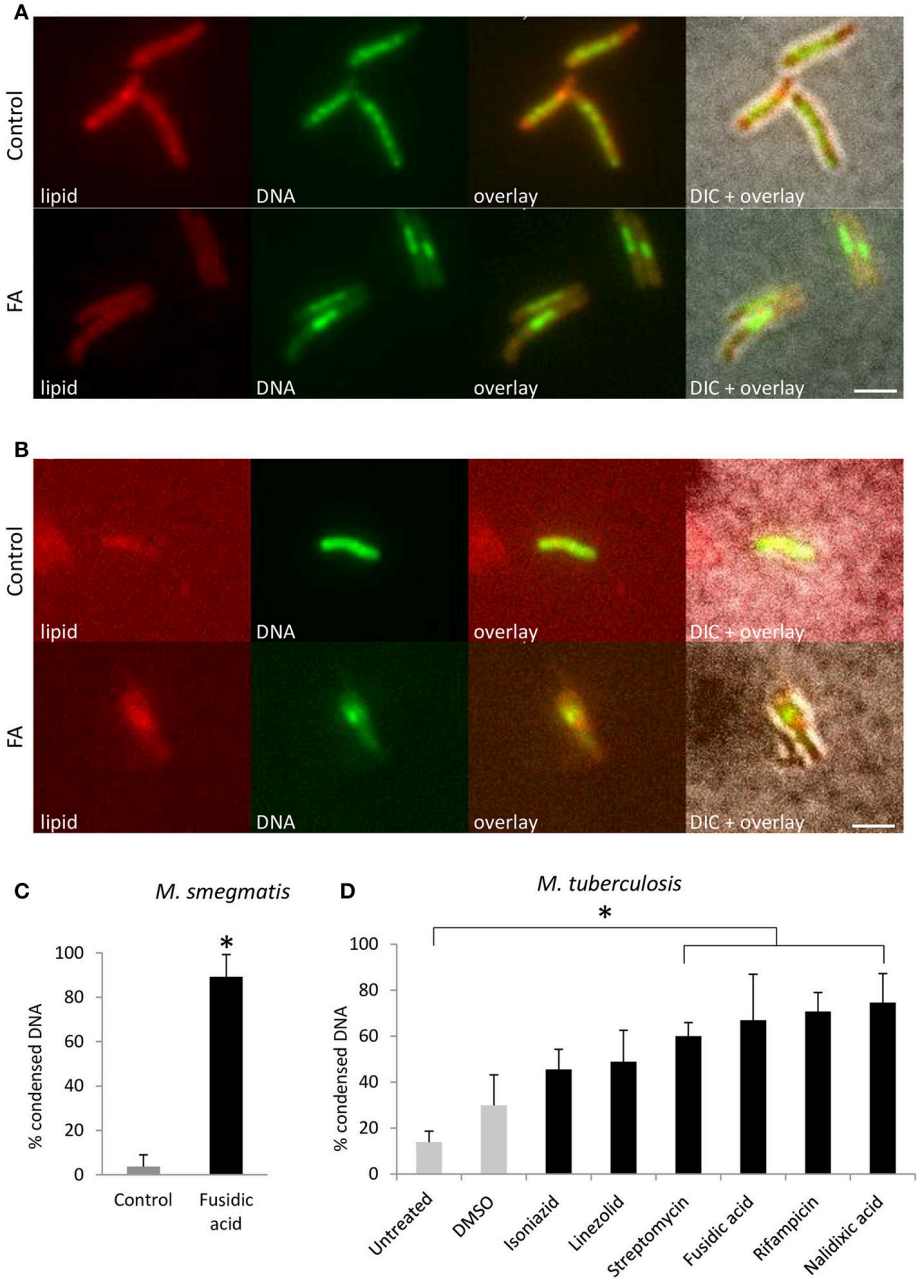
DNA condensation is a generic response to antibiotic-induced stress in *Mycobacterium*. Fluorescent microscopy images of lipid and DNA patterns in *M. smegmatis* mc^2^155 **(A)** and *M. tuberculosis* mc^2^6030 **(B)** in control and FA treatment conditions. Lipids stained with BODIPY (red), DNA stained with Hoechst 33342 (green), an overlay of the two fluorescent signals and the overlay with the bright-field image (DIC). **(C)** Average bacteria with condensed DNA distribution patterns in *M. smegmatis*, in control (gray bar) and after 1 h FA-treatment (black bar, bars represents mean ± standard error, *n* = 3, *P < 0.05). **(D)** *M. tuberculosis* mc^2^6030 was exposed to various antibiotics to target different cellular processes. The incidence of DNA condensation was quantified for untreated, DMSO treated as a control (gray bars) and antibiotic treated (black bars) *M. tuberculosis* mc^2^6030 cultures. Per condition, the percentage of bacilli with condensed DNA is displayed, which was based on three measurements of *n* ≥ 20 bacteria per condition. Treatment with streptomycin, fusidic acid, rifampicin and nalidixic acid increased the percentage bacteria with condensed DNA significantly (bar represents mean ± standard error, *P < 0.05). Scale bar represents 2 μm.

To gain further insights into the prevalence of nucleoid aggregation upon stress, *M. smegmatis* mc^2^155 and *M. tuberculosis* mc^2^6030 were treated with antibiotics or mock-treated, and localization of DNA was categorized and quantified by FM. During early log phase, DNA of untreated *M. smegmatis* appeared distributed across the cell in a distinctive pattern, forming a chain of small nucleoids. However, more than 90% of the bacteria condensed their DNA into a single nucleoid after FA treatment (Figure 2A). Similarly, *M. tuberculosis* mc^2^6030 subjected to the same experimental conditions condensed their DNA into a single nucleoid (Figure 2B). For *M. smegmatis* mc^2^155 cultures treated with FA or mock-treated, DNA localization was categorized and quantified after 1 h of antibiotic stress, more than 80% of the bacteria condensed their nucleoid (Figure 2C). Thus, within 1 hour of antibiotic-induced stress, the DNA in mycobacteria rearranges and condenses into a single clump.

To evaluate whether DNA condensation in mycobacteria is a generic response to antibiotic-induced stress, *M. tuberculosis* mc^2^6030 was exposed to a variety of antibiotics that hamper DNA replication, transcription, translation, and cell wall synthesis (Figure 2D). Treatment with streptomycin, fusidic acid, rifampicin and nalidixic acid resulted in a significantly increased fraction of bacilli with condensed DNA, indicating that DNA condensation is a generic response to antibiotic-induced stress in *M. tuberculosis*.

**Figure 3:**
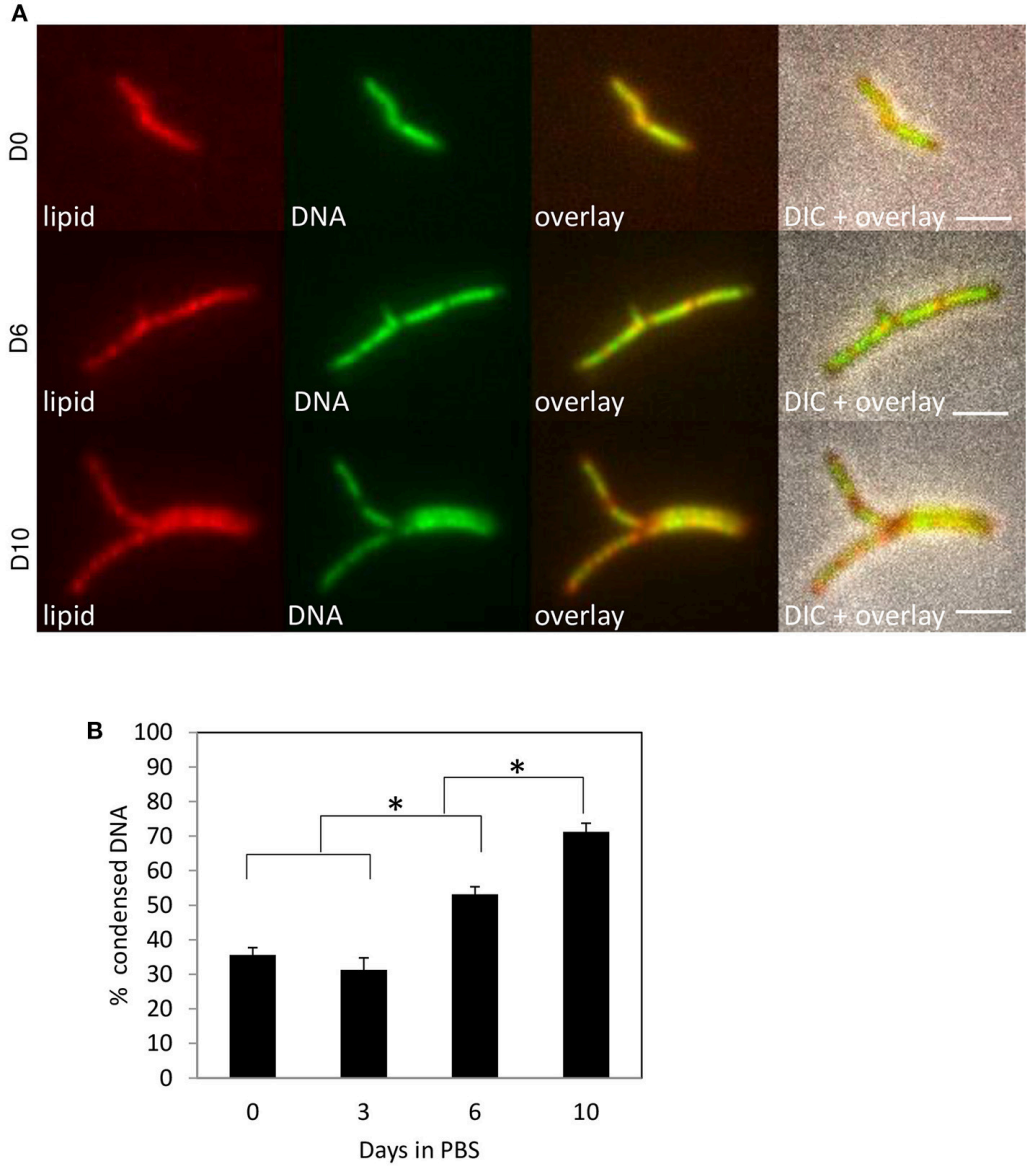
*M. tuberculosis* condenses DNA during starvation. *M. tuberculosis* mc^2^6030 was cultured in ADC-supplemented Middlebrook 7H9 medium before being starved in PBS. Lipid distribution and DNA localization was imaged using Nile Red (red) and Hoechst (green) respectively at day 0, 6, and 10 **(A)** and percentage bacteria with condensed DNA was quantified at day 0, 3, 6, and 10. Values represent mean percentage bacteria with condense DNA ± standard error, pooled data of 2 measurements, *P < 0.05 **(B)**. Scale bars represent 2 μm.

To evaluate if DNA condensation might occur under conditions of stress caused by agents other than antibiotics, nutrient starvation was carried out by culturing *M. tuberculosis* and *M. smegmatis* in phosphate-buffered saline (PBS) after regular culturing. These culturing conditions need to be maintained for 14 days or longer to induce quiescence in *M. tuberculosis* (Gengenbacher et al., 2010), and several hours to starve *M. smegmatis* (Wu et al., 2016b,c). At different time points, the incidence of DNA condensation was evaluated (Figure 3 and Supplemental Figure 2). Nucleoid condensation was apparent in *M. smegmatis* after 1–4 h of culturing in PBS. For *M. tuberculosis*, a slow growing bacterium, which is extremely resistant to starvation, a gradual increase in DNA condensation was visible after 10–14 days. Interestingly, the nucleoid was relocated to the poles of *M. smegmatis* after 24 h, whereas this condensation pattern was evident neither for *M. tuberculosis*, nor for *M. smegmatis* treated with FA. In summary, our experiments demonstrate DNA condensation in response to starvation- and antibiotic-induced stress, demonstrating that DNA condensation is a generic response to stress in mycobacteria.

### DNA condensation responses are limited to viable mycobacteria

Condensation of DNA could be an indication of cell death, similar to eukaryotic apoptosis and it has indeed been reported that that nucleoid-condensation accompanies cell death in *E. coli* (Dwyer et al., 2012), whereas others have reported that DNA condensation upon stress is reversible, suggesting that it is restricted to viable bacteria (Levin-Zaidman et al., 2000).

To determine whether DNA condensation is reversible in mycobacteria, *M. tuberculosis* mc^2^6030 cultures were treated with FA for 1 h and washed to remove the antibiotic, followed by regular culturing for 24 h (Supplemental Figure 3). The incidence of DNA condensation increased until 6 h after treatment, followed by a gradual reduction. This decrease could not be attributed to the progression of cell division because the duplication time of *M. tuberculosis* is ~20 h. Thus, DNA condensation is a reversible process in mycobacteria.

**Figure 4:**
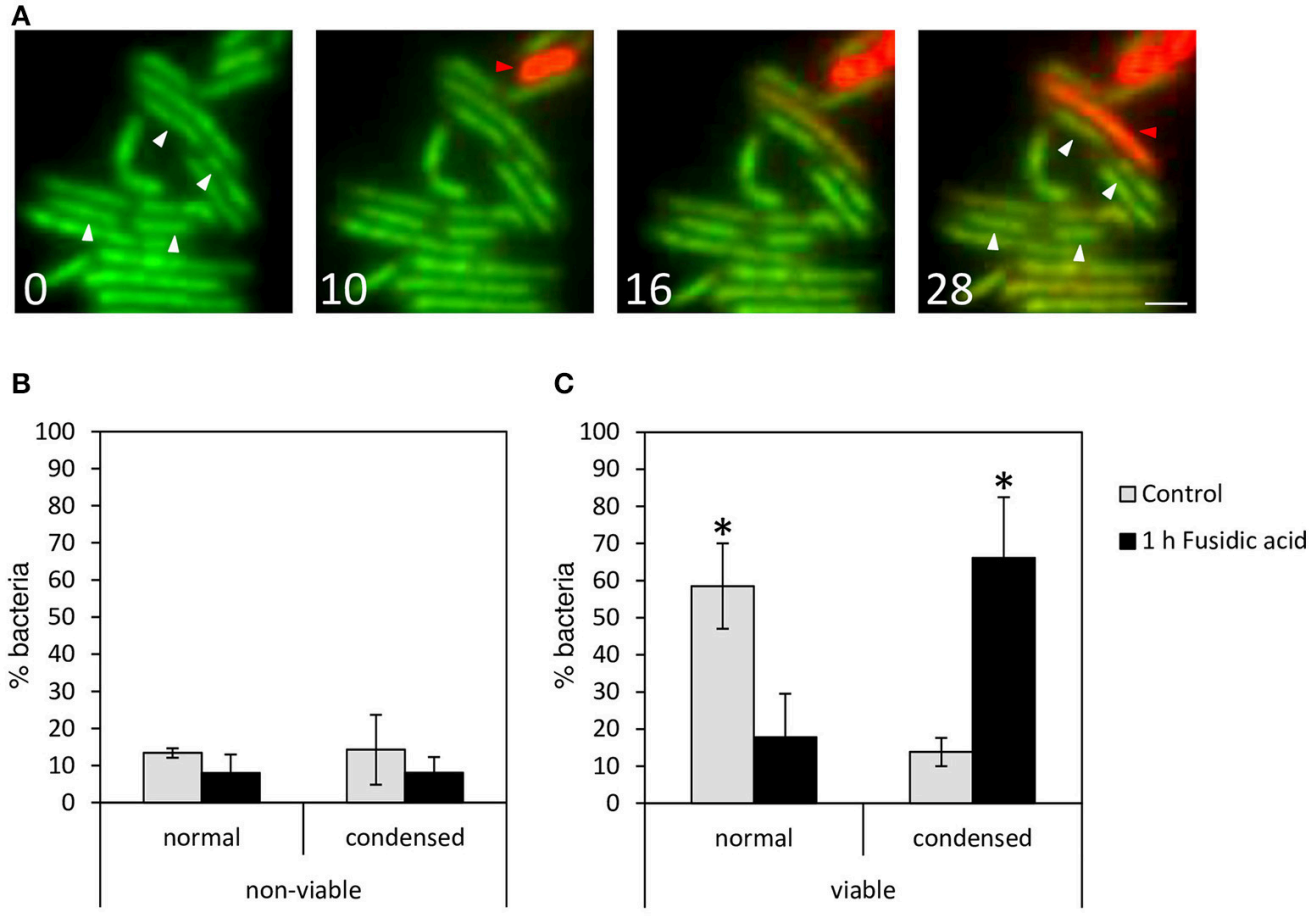
*Mycobacteria* with condensed DNA are viable. To evaluate the viability of treated *M. smegmatis* with condensed DNA at single cell resolution, a bacterial viability staining was applied to live bacteria 5 min after FA treatment and imaged at *t* = 0, 10, 16, and 28 min (**A**; see also movie in Supplemental Figure 3). Live bacteria (green) condense their DNA (white arrowheads), and in red, dying bacteria with dispersed DNA (red arrowheads). Scale bar represents 1 μm. **(B,C)** Quantification of *M. tuberculosis* mc^2^6030 control (gray bars) and after 1 h treatment with FA (black bars). Bacteria stained with Syto9- and PI-fluorescence were fixed with PFA and images were generated by confocal fluorescence microscopy. DNA distribution of **(B)** nonviable and **(C)** viable *M. tuberculosis* mc^2^6030 bacteria were quantified as dispersed (normal) and condensed. Bars represent mean ± standard error. Data were pooled from 3 measurement sessions and occurrence of DNA condensation in viable control and FA-treated cells was compared with a Chi2-test, *P < 0.05.

To support these findings, we evaluated the viability of mycobacteria with condensed nucleoids after FA treatment at single-cell resolution using a viability staining, which allows distinguishing between viable and inviable bacteria based on the integrity of the cell wall. Live-cell imaging of *M. smegmatis* was started 5 min after FA addition, when the nucleoid is uncondensed. Differences in nucleoid localization became visible after 10 min, and the first bacteria converted from viable (green) to inviable (red) after 16 min (Figure 4A, movie in Supplemental Figures 3, 4). However, while bacteria that condensed their DNA survived the entire duration of the experiment (white arrowheads in Figure 4A), the dying bacteria did not condense their nucleoid (red arrowheads). To support this observation, the incidence of DNA condensation was evaluated in FA-treated and control *M. tuberculosis* mc^2^6030 cultures that were fixed after the viability staining (Figures 4B,C). One hour after FA treatment, the percentage of viable bacteria with condensed DNA increased significantly, whereas the percentage of nonviable bacteria with condensed DNA did not differ between the untreated and FA-treated cultures.

In summary, DNA condensation upon antibiotic-induced stress is a reversible process, limited to viable mycobacteria, and therefore, it might be part of the survival strategy of *M. tuberculosis* under stress conditions, rather than a manifestation of cell death.

### Altered DNA distribution is DNA condensation

**Figure 5:**
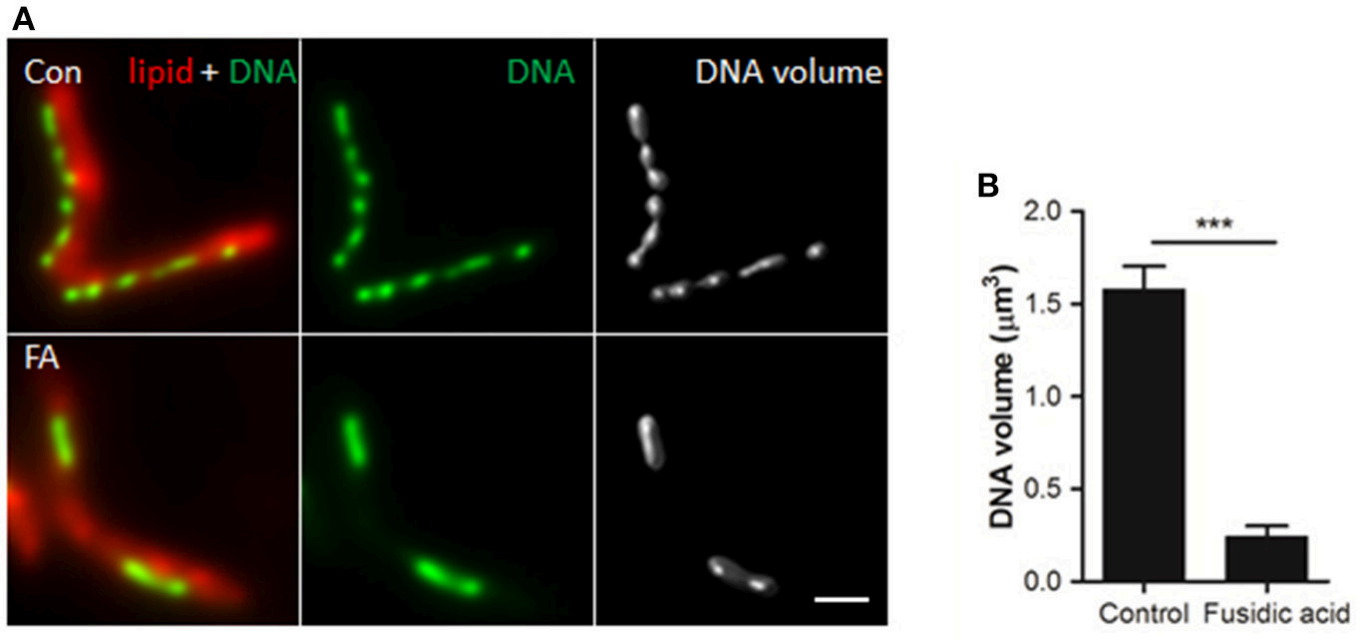
DNA volume decreases in response to antibiotic-induced stress. **(A)** Deconvolved widefield fluorescence microscopy images of lipid (Nile Red) and DNA (DAPI) patterns in (un)treated M. smegmatis mc2 155. Deconvolved DAPI signal was used to compute DNA volume. Scale bar represents 1 μm. **(B)** Quantification of DNA volume. Bars represent mean ± standard deviation of 3 independent experiments (*n* = 60 bacteria per condition). ***P < 0.0005.

To determine whether the observed DNA redistribution is a result of condensation or rearrangement, the average 3D volume of the DNA clusters was measured in *M. smegmatis* cultures incubated with or without FA for 1 h (Figure 5A). We observed a significantly larger average cluster volume in the control vs. the FA treated bacteria (1.58 and 0.24 μm^3^, respectively Figure 5B), confirming DNA condensation under the latter experimental condition.

### SMCs are not involved in DNA condensation upon stress

**Figure 6:**
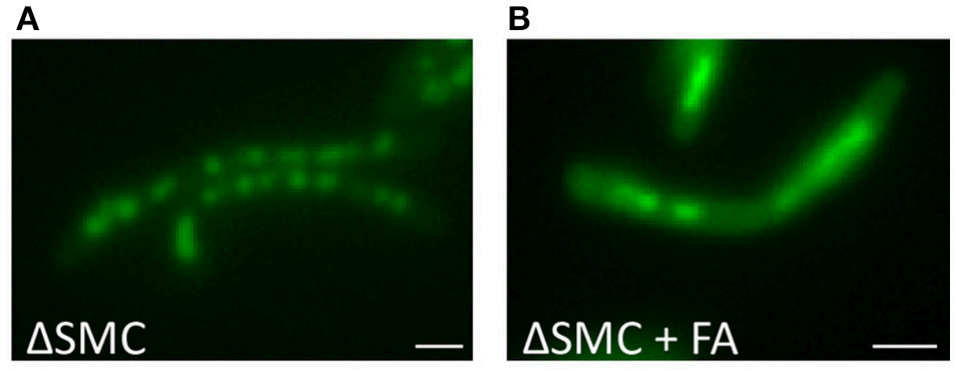
DNA condensation is independent of SMC. DNA stained with Hoechst of Δ SMC triple mutant *M. smegmatis* mc^2^6 cultures **(A)** untreated or **(B)** treated with for 1 h with Fusidic Acid (FA). Bar represents 1 μm.

As our experiments suggested that DNA condensation might be a survival strategy, we speculated that interference with this process would sensitize bacteria to antibiotic treatments. Both DNA condensation and de-condensation are likely to be essential for the recovery from stress and progress of cell division (Eskandarian et al., 2017). DNA-binding proteins are probable participants in this process, and several DNA-binding proteins involved in regulating the DNA condensation state have already been identified. In addition to the nucleoid associated proteins described above, the structural maintenance of chromosomes (SMC) proteins are highly conserved factors involved in chromosome organization and compaction in eukaryotes and most bacteria (Sullivan et al., 2009). There are three SMC paralogs in *M. smegmatis*. MSMEG_2423 is conserved in all mycobacterial species and shares homology with the SMC from gram-positive bacteria. EptC and MSMEG_0370 are MukB like proteins and expression of EptC interferes with the segregation of plasmids with pAL5000 origin of replication by manipulating plasmid DNA topology (Panas et al., 2014). To investigate whether these SMCs play a role in FA dependent nucleoid condensation, the triple SMC deletion knock-out mc^2^3449 was compared to its isogenic strain mc^2^155 before and after FA treatment. Surprisingly, the DNA in the triple deletion knock-out mc^2^3449 still condensed into nucleoids, similar to the control strain. More importantly, DNA condensation was clearly visible after FA treatment, suggesting that these SMCs are redundant for this phenotype (Figure 6).

### Blocking acetylation after DNA condensation kills mycobacteria

Ideally, DNA condensation or de-condensation could be manipulated by inhibitors specific to bacteria and neutral to humans. As bacteria and eukaryotes have distinct histone-like proteins, these proteins could represent a selective antibiotic-target. Multiple different histone-like proteins affect the organization of the bacterial genome. For instance, Rv3852 is a histone-like protein involved in several pleiotropic phenotypic changes, including DNA compaction, as *M. smegmatis* overexpressing Rv3852 show a dispersed genome localization (Ghosh et al., 2013). Recently, it was demonstrated that acetylation of a histone-like protein termed MtHU by the enzyme Eis reduces its DNA-binding capacity, leading to decompaction of DNA (Ghosh et al., 2016). Thus, the DNA-binding properties of mycobacterial histone-like proteins are at least partly regulated by posttranslational modifications such as acetylation. We therefore investigated whether interfering with acetylation affects the condensation state of the DNA.

**Figure 7:**
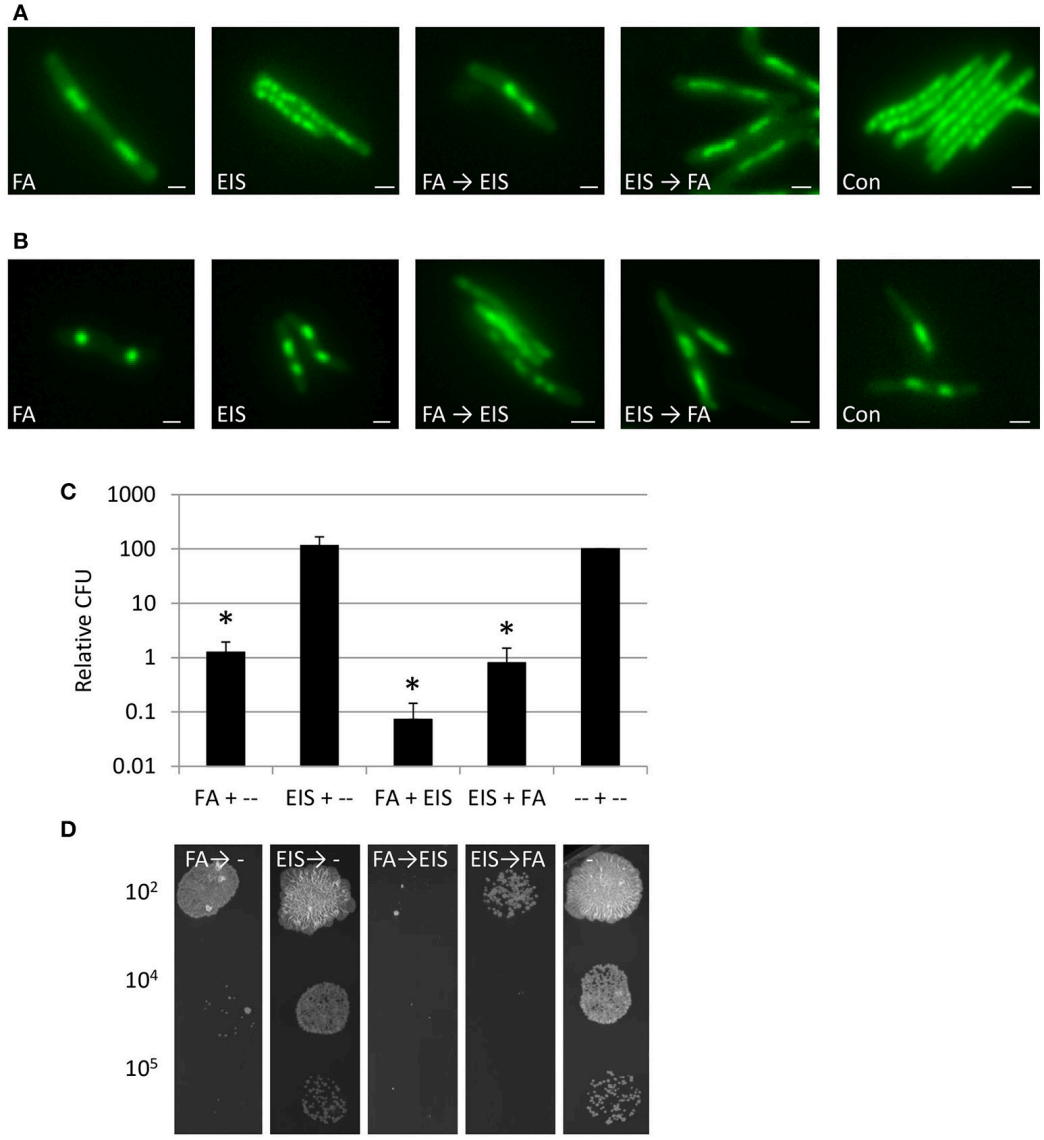
Inhibition DNA acetylation after condensation DNA, improves killing *M. smegmatis*. **(A)** Fluorescence microscopy of DNA of *M. smegmatis* treated with FA, compound EIS 1a*, inhibiting the Eis enzyme (Eis) or FA and subsequently Eis inhibitor (FA → EIS), the reverse order (EIS → FA) or control (Con) (Nile Red stained as counterstaining is presented in Supplemental Figure 5A). **(B)** Similar set-up as in **(A)** imaged after 2 days of incubation (Nile Red stained as counterstaining is presented in Supplemental Figure 5B). **(C)** Relative Colony Forming Units (CFU) based on untreated controls at 6 days after incubation in liquid antibiotic containing medium and plated on antibiotic free plates (relative CFU is calculated based on untreated controls and average of 3 independent experiments with standard error and * for significant differences to control *P* < 0.05). **(D)** Representative colonies from 10 μl 7H9 medium at a dilution of 10^2^, 10^4^, or 10^5^ with FA, Eis inhibitor or antibiotic combinations or control after incubation for 6 days in 7H9 and subsequently grown on 7H10 plate without antibiotics. Bars represent 1 μm.

Several highly potent and selective Eis inhibitors have been developed (Garzan et al., 2016a,b, 2017; Willby et al., 2016), suppressing the aminoglycoside acetylation activity of Eis *in vitro* and in *M. tuberculosis* (Chen et al., 2012) cultures. The pyrrolo[1,5-*a*]pyrazine-based Eis inhibitor 1a^*^ was shown to inhibit kanamycin acetylation biochemically, biologically and structurally (Garzan et al., 2017; Green et al., 2018), and therefore we chose to examine whether this inhibitor could block DNA condensation. At MIC_50_, Eis inhibitor 1a^*^ treated *M. smegmatis* mc^2^155 cultures did not reveal DNA condensation (Figure 7A, Supplemental Figure 5). In addition, at 2 h of incubation, Eis inhibitor 1a^*^ did not affect FA-induced DNA-relocalisation, irrespective of whether it was added before (EIS → FA) or after FA (FA → EIS), indicating that inhibiting EIS does not influence the FA induced DNA condensation. As the bactericidal effect of Eis inhibition might take a few days to develop, effects of the combination treatment might be likewise delayed. Therefore, DNA localisation was monitored for several days. Untreated and EIS 1a^*^ treated bacilli grown for 2 days (Figure 7B, Supplemental Figure 5) displayed condensed DNA similar to that observed under starvation conditions in PBS (Supplemental Figure 2), which can be attributed to the high OD of these cultures. However, bacilli treated first with FA for 1 h and subsequently by Eis inhibitor 1a^*^ for 1 h (FA → EIS) showed an amorphous DNA distribution at this time point, whereas bacteria treated in the reverse order (EIS → FA), or by FA alone, still displayed condensed DNA. These results suggest that acetylation is involved in recovery of the structural organization of the nucleoid. The amorphous DNA distribution could have an effect on the survival of mycobacteria, and thus growth of cultures treated with FA, Eis inhibitor 1a^*^, or the successively administrated combinations was monitored by determining CFU on antibiotic free 7H10 plates.

Colony forming ability of cultures treated with FA → EIS 1a^*^ on day 2, 3, and 6 was significantly reduced (Supplemental Figure 5), as compared to those treated with FA alone and with EIS inhibitor 1a^*^ → FA [Figures 7C,D; Supplemental Figure 5 (*P* < 0.05)]. Thus, interfering with acetylation of DNA-binding proteins such as MtHU after antibiotic treatment results in increased cytotoxicity and the order of administration of the successive FA → EIS 1a^*^ treatment is critical. The amorphous DNA distribution detected in bacteria cultured under these conditions indeed correlates with reduced survival. In summary, our results suggest that the EIS inhibitor can interfere with the recovery after DNA condensation, which renders mycobacteria more vulnerable to antibiotic treatments.

## Discussion

DNA condensation is thought to preserve genome integrity in bacteria (Almirón et al., 1992; Martinez and Kolter, 1997; Wolf et al., 1999; Gordon and Wright, 2000; Smith et al., 2002; Qu et al., 2013; Shechter et al., 2013; Badrinarayanan et al., 2015) and in mycobacteria condensed DNA is detected as foci in dividing bacteria (Santi et al., 2013), in log vs. stationary phase (Vaubourgeix et al., 2015), after starvation in PBS for *M. tuberculosis* (Figure 3) and for *M. smegmatis* (Wu et al., 2016c), and after treatment with different antibiotics (Figure 2). Condensation of DNA thus appears to be a generic response to various stress conditions and our study extend this conclusion to *M. tuberculosis* and *M. smegmatis*, which condense their DNA in response to stress. Recently, cryo-electron tomography demonstrated that viral phage-infected bacteria assemble the membrane-wrapped nucleus-like structures (Chaikeeratisak et al., 2017), which are different from the membrane-less DNA condensates described here. The authors demonstrated that the compact DNA is dynamic and allows for viral DNA replication. Our results show that DNA condensation in mycobacteria is dynamic, reversible and unlikely to be associated with cell death, as suggested by others (Dwyer et al., 2012). Furthermore, the involvement of acetylation in the DNA (de)condensation process we detected, may resemble mammalian regulation of chromatin compaction that is partly driven by histone acetylation (Kouzarides, 2007). Interestingly, even though conclusive data are not available, it has been suggested that the condensed heterochromatin in mammalian cells is more resistant to double stranded breakage induction, as compared to euchromatin, and associated with increased radio-resistance (Falk et al., 2010; Storch et al., 2010). DNA compaction may thus represent a widely preserved response to stress, which could be of benefit in the early evolutionary history of life when harsh environmental conditions may have continuously threatened genomic integrity.

The mechanism driving DNA condensation is not yet understood, but our live-cell imaging experiments demonstrate that it is a relatively fast process, as condensation occurred within 10 min after stress-induction. Others have suggested that condensation may be a product of entropic forces, molecular crowding and actions of NAPs or other DNA binding proteins (de Vries, 2010; Shendruk et al., 2015) and its fast nature observed here is in agreement with at least the first two of these suggestions. However, our results demonstrate that the participation of SMCs is unlikely (Figure 6) like the acetylation of DNA binding proteins. On the other hand, NAPs could be involved in recovery after condensation as overexpression of DNA binding proteins resulted in de-compaction of DNA (Ghosh et al., 2013, 2016) and the recovery after condensation can be inhibited by blocking acetylation in general, and presumably more specifically of DNA binding histone-like proteins.

New strategies for drug administration are urgently needed, especially to treat the quiescent and multidrug-resistant *M. tuberculosis* strains. The ongoing clinical trials mostly focus on identifying high-efficacy treatment regimens based on combining new (bedaquiline and delamanid Diacon et al., 2009; Gler et al., 2012) and old antibiotics. Accordingly, the current WHO recommendation for treatment of multi-drug resistant tuberculosis in some patients with limited options includes a combination therapy or concomitant use of multiple drugs (Matteelli et al., 2016). Our results suggest a more refined, conceptually novel strategy where a stress-inducing (antibiotic) agent is first used to provoke a protective response in the pathogen and subsequently the cytotoxic effect is potentiated by inhibiting the recovery from this protective response. Accordingly, the order of administration of these drugs is crucial for the treatment outcome. Indeed, we found that while exposing bacteria to the acetylation EIS inhibitor 1a^*^ after FA-induced DNA condensation dramatically enhanced the cytotoxicity, the same combination failed to achieve similar effects when the inhibitor was administered before DNA condensation. Thus far, a comparable approach has not been considered and the only drug that binds and condenses bacterial chromosomes to kill both Gram-negative and Gram-positive species is the antimicrobial polyhexamethylene biguanide (PHMB) (Chindera et al., 2016).

The cytotoxicity analysis presented here focused on the activity of EIS 1a^*^ inhibitor in *M. smegmatis* which in combination potentiated the efficacy of fusidic acid by 10 fold. For the application as an effective antimicrobial strategy the decrease in CFU should be higher and clearly effective in *M. tuberculosis*. However, our preliminary experiments showed that structurally unrelated sulphonamide acetylation inhibitor is not active in *M. tuberculosis* (data not shown). Therefore, our ongoing experiments aim at testing the activity of EIS 1a^*^ and a library of its derivatives in *M. tuberculosis*. We believe that *M. tuberculosis*-optimized inhibitors could have immediate impact on rational design of combination treatment strategies exploiting protective DNA condensation as a new Achilles' heel of mycobacteria.

## Author contributions

EMS, ER, PK, and NW wrote the paper. EMS, ES, KG, SK, AEG, and HV performed experiments. JK and PK performed live imaging. AG, HN, KG, and SG-T generated Eis inhibitor. JR performed statistical analysis. EMS, ES, and NW conceived and designed experiments.

### Conflict of interest statement

The authors declare that the research was conducted in the absence of any commercial or financial relationships that could be construed as a potential conflict of interest.

## Supplementary material

Supplemental Figure 1 DNA condensation imaged with EM and CLEM. Tomogram slices containing a semi-thick (300 nm) section of *M. smegmatis* control **(A)** or FA treated **(B)**. Stacks are artificial color coded based on electron-density, with in red e-dense ribosome-like structures, in green DNA clusters, top section (TEM) and the segmentations of the tomograms separate from the TEM image (tomo). Scale bars represent 500 nm and A′ or B′ movie focusing through the section, depicting different layers. **(C)** Combined Light and Electron Microscopy images of a small group of intact, PFGA fixed *M. smegmatis* treated with FA for 1 h and stained with BODIPY (red, lipid), DAPI-Hoechst (green, DNA arrowheads indicate clustered DNA), the combination of DNA and Lipid and combinations of EM and DNA and Lipid, bar represents 2 μm. **(D)** high magnification CLEM and **(D**′**)** EM image of fixed *M. smegmatis* from **(C)** demonstrating e-lucent areas in the bacteria that correspond to the DNA clusters (arrowheads). Bar represents 2 μm.

Supplemental Figure 2 *M. smegmatis* condenses DNA during starvation. *M. smegmatis* was cultured in ADC-supplemented Middlebrook 7H9 medium before being transferred to PBS. Bacteria were starved in this medium for 0, 1, 4, and 24 h. Lipid distribution (Nile Red) and DNA localization (Hoechst in green) and overlay with DIC detected at the different time points **(A)**. Genome localization was quantified and categorized in dispersed, condensed and polar at 0, 1, 4, 24 h **(B)**. Bars represent mean ± standard error *n* = 2, based on >500 bacteria. Scale bars represent 2 μm.

Supplemental Figure 3 *M. tuberculosis* condensation is reversible. The reversibility of DNA condensation after antibiotic treatment was evaluated. **(A)** At time points pre- and post-treatment of *M. tuberculosis* mc^2^6030 with FA for 1 h, the incidence of DNA condensation was quantified in fixed samples stained with DAPI using confocal fluorescence microscopy. Values represent mean ± standard error based on 8 measurements of *n* ≥ 20 bacteria per time point, originating from 2 independent experiments ^*^*P* < 0.05. **(B)** DNA in response to antibiotic-induced stress, schematically illustrated, with green resembling DNA-localization. **(C)** Live cell imaging combined with bacterial viability staining applied to live *M. smegmatis* 5 min after FA treatment and imaged at *t* = 0 till 28 min with 5 min intervals (see also separate movie file and stills in Figure 4).

Supplemental Figure 4 Live cell imaging of FA treated *M. smegmatis*. Two *M. smegmatis* bacteria are imaged 5 min after FA treatment, stained with bacterial viability staining and imaged at *t* = 0 till 38 min with 2 min intervals **(A)** with stills in **(B)**.

Supplemental Figure 5 Inhibition DNA acetylation after condensation DNA, does not prevent DNA condensation but improves killing *M. smegmatis*. **(A)** Fluorescence microscopy of DNA (green) and lipids with Nile Red stained as counterstaining (red) of *M. smegmatis* cultures treated with FA (FA), compound Eis 1a^*^, inhibiting the Eis enzyme (EIS) or FA and subsequently Eis inhibitor (FA → EIS), the reverse order (EIS → FA) or control (Con). **(B)** Similar set-up as in **(A)** imaged after 2 days of incubation in antibiotics. **(C)** Average CFU at 2 h,1, 2, 3, and 6 days after incubation in liquid antibiotic containing medium and plated on antibiotic free plates (average of 3 measurements of 3 independent experiments with standard error). **(D)** A one-way ANOVA was performed on log transformed CFU data per time point. Subsequently Student-Newman-Keuls test was applied to identify subsets of conditions with similar effects on CFU (*P* < 0.05). Bars represent 1 μm.
