# Supplementary figures and images for: Interfering With DNA Decondensation as a Strategy Against Mycobacteria
Source: Front Microbiol. 2018 Sep 5;9:2034. doi: 10.3389/fmicb.2018.02034 (PMC6135046; doi:10.3389/fmicb.2018.02034)

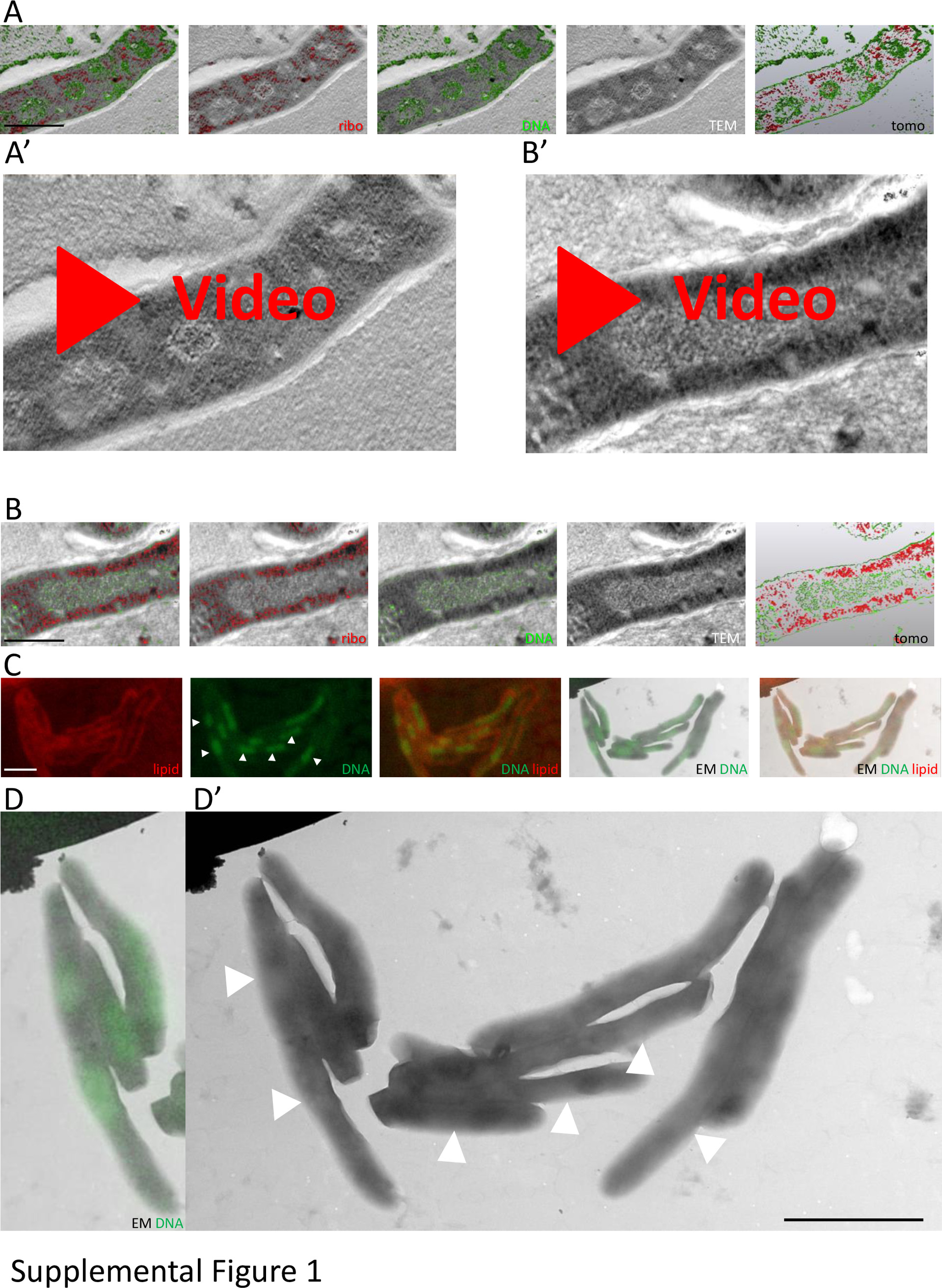

Supplement: Supplemental Figure 1 — DNA condensation imaged with EM and CLEM. Tomogram slices containing a semi-thick (300 nm) section of M. smegmatis control (A) or FA treated (B). Stacks are artificial color coded based on electron-density, with in red e-dense ribosome-like structures, in green DNA clusters, top section (TEM) and the segmentations of the tomograms separate from the TEM image (tomo). Scale bars represent 500 nm and A′ or B′ movie focusing through the section, depicting different layers. (C) Combined Light and Electron Microscopy images of a small group of intact, PFGA fixed M. smegmatis treated with FA for 1 h and stained with BODIPY (red, lipid), DAPI-Hoechst (green, DNA arrowheads indicate clustered DNA), the combination of DNA and Lipid and combinations of EM and DNA and Lipid, bar represents 2 μm. (D) high magnification CLEM and (D′) EM image of fixed M. smegmatis from (C) demonstrating e-lucent areas in the bacteria that correspond to the DNA clusters (arrowheads). Bar represents 2 μm. [file Image_1.JPEG]

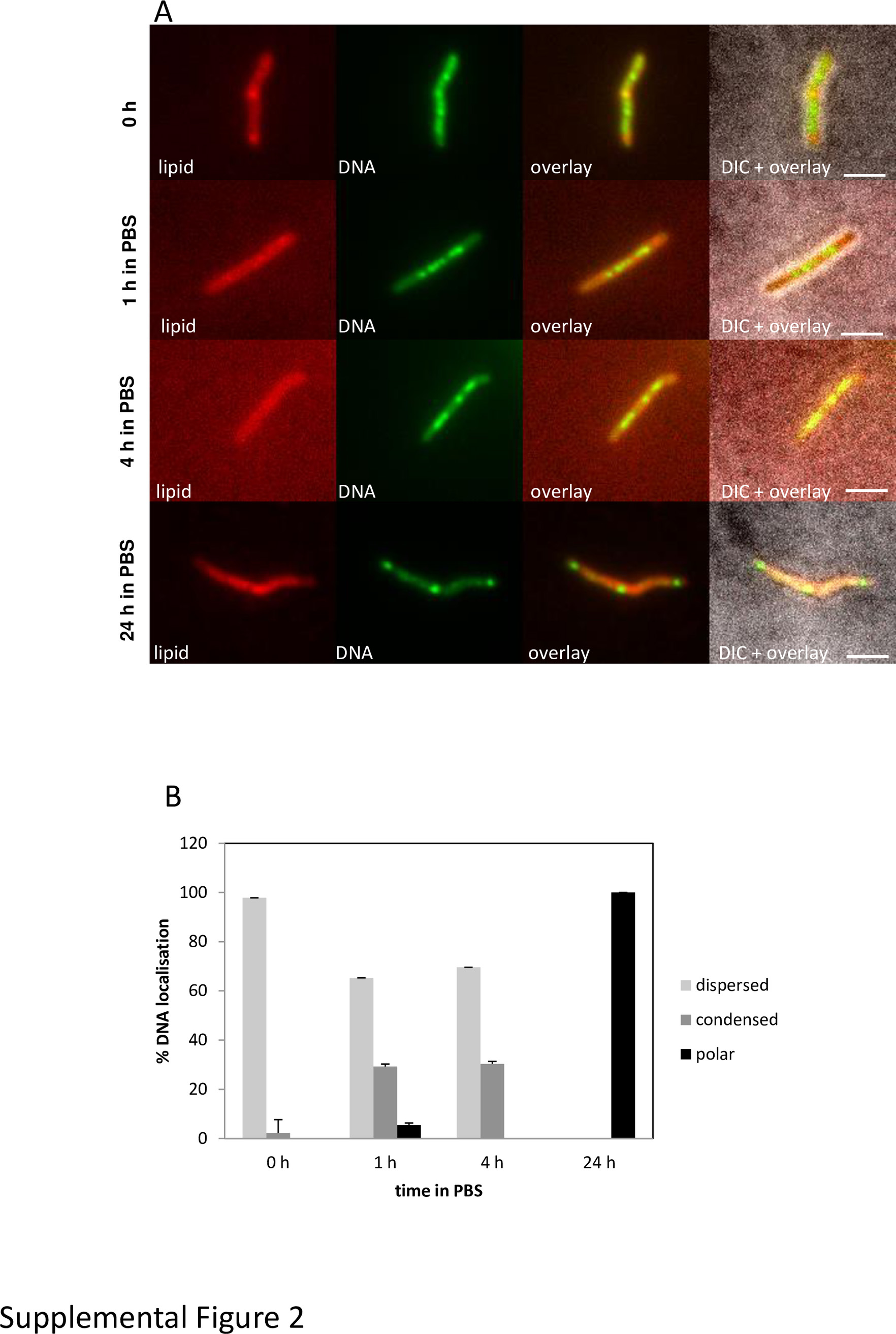

Supplement: Supplemental Figure 2 — M. smegmatis condenses DNA during starvation. M. smegmatis was cultured in ADC-supplemented Middlebrook 7H9 medium before being transferred to PBS. Bacteria were starved in this medium for 0, 1, 4, and 24 h. Lipid distribution (Nile Red) and DNA localization (Hoechst in green) and overlay with DIC detected at the different time points (A). Genome localization was quantified and categorized in dispersed, condensed and polar at 0, 1, 4, 24 h (B). Bars represent mean ± standard error n = 2, based on >500 bacteria. Scale bars represent 2 μm. [file Image_2.JPEG]

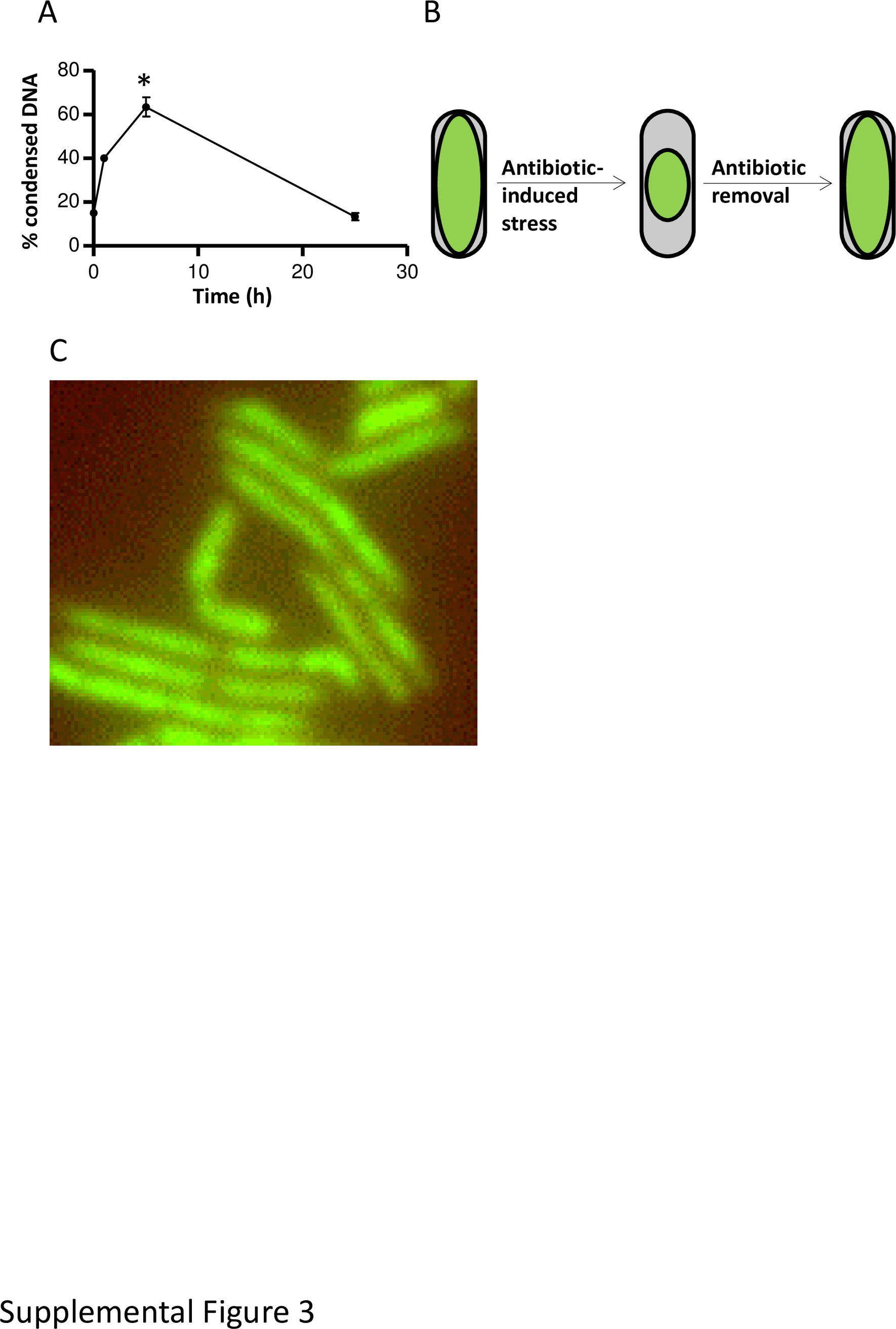

Supplement: Supplemental Figure 3 — M. tuberculosis condensation is reversible. The reversibility of DNA condensation after antibiotic treatment was evaluated. (A) At time points pre- and post-treatment of M. tuberculosis mc26030 with FA for 1 h, the incidence of DNA condensation was quantified in fixed samples stained with DAPI using confocal fluorescence microscopy. Values represent mean ± standard error based on 8 measurements of n ≥ 20 bacteria per time point, originating from 2 independent experiments *P < 0.05. (B) DNA in response to antibiotic-induced stress, schematically illustrated, with green resembling DNA-localization. (C) Live cell imaging combined with bacterial viability staining applied to live M. smegmatis 5 min after FA treatment and imaged at t = 0 till 28 min with 5 min intervals (see also separate movie file and stills in Figure 4). [file Image_3.JPEG]

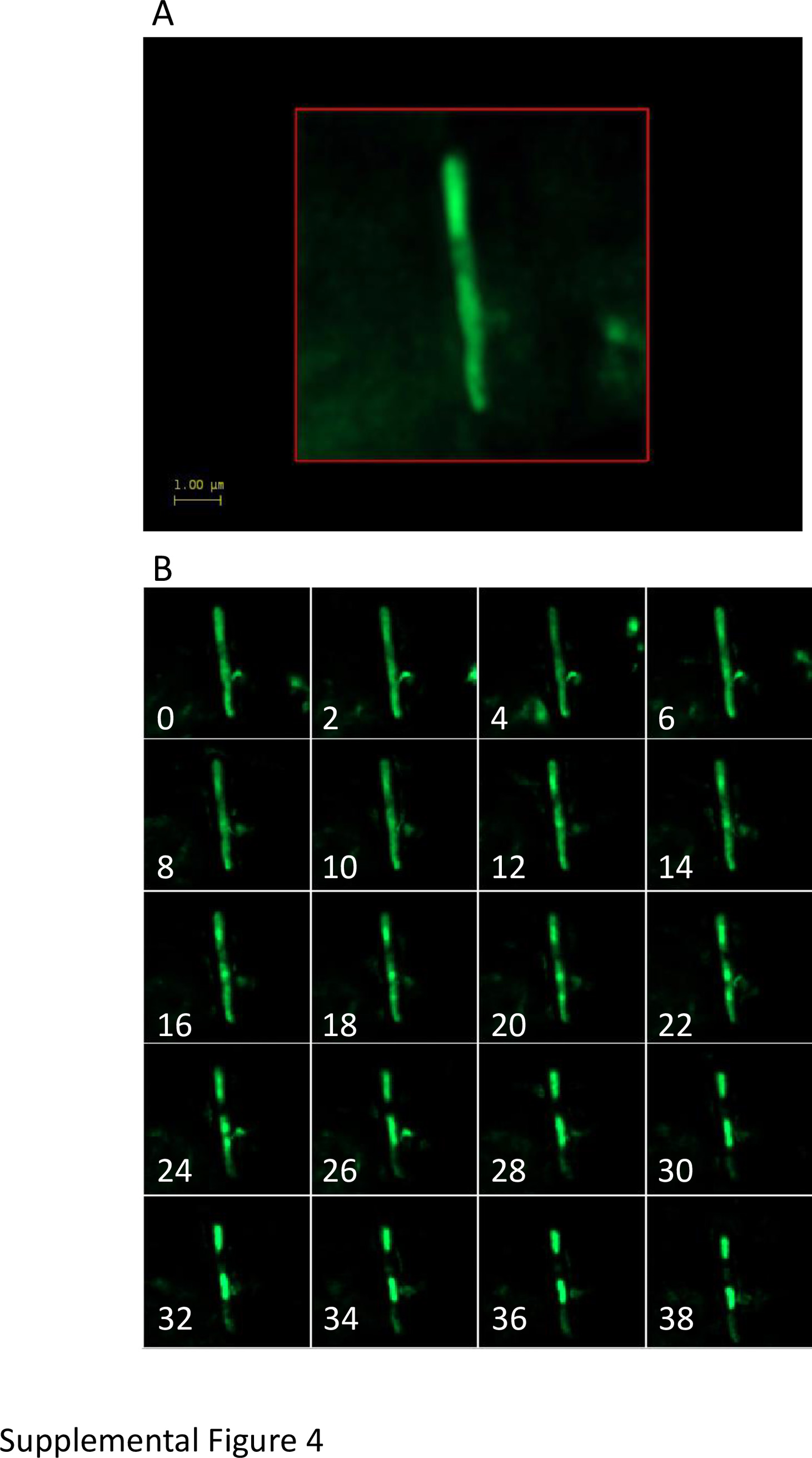

Supplement: Supplemental Figure 4 — Live cell imaging of FA treated M. smegmatis. Two M. smegmatis bacteria are imaged 5 min after FA treatment, stained with bacterial viability staining and imaged at t = 0 till 38 min with 2 min intervals (A) with stills in (B). [file Image_4.JPEG]

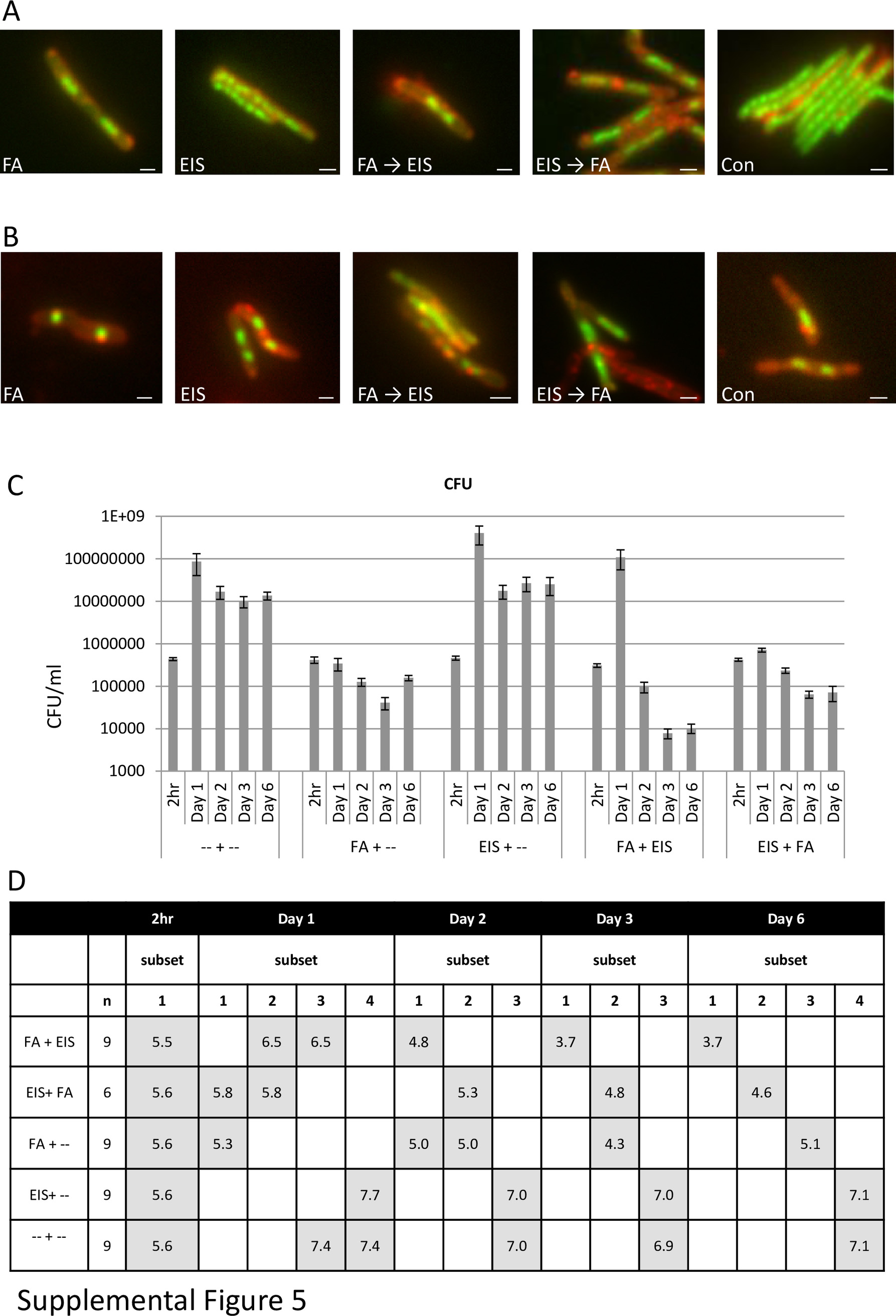

Supplement: Supplemental Figure 5 — Inhibition DNA acetylation after condensation DNA, does not prevent DNA condensation but improves killing M. smegmatis. (A) Fluorescence microscopy of DNA (green) and lipids with Nile Red stained as counterstaining (red) of M. smegmatis cultures treated with FA (FA), compound Eis 1a*, inhibiting the Eis enzyme (EIS) or FA and subsequently Eis inhibitor (FA → EIS), the reverse order (EIS → FA) or control (Con). (B) Similar set-up as in (A) imaged after 2 days of incubation in antibiotics. (C) Average CFU at 2 h,1, 2, 3, and 6 days after incubation in liquid antibiotic containing medium and plated on antibiotic free plates (average of 3 measurements of 3 independent experiments with standard error). (D) A one-way ANOVA was performed on log transformed CFU data per time point. Subsequently Student-Newman-Keuls test was applied to identify subsets of conditions with similar effects on CFU (P < 0.05). Bars represent 1 μm. [file Image_5.JPEG]
